# Meckel’s Diverticulum as a Cause of Small Bowel Obstruction Complicated with Gangrene in the Third Trimester of Pregnancy: A Case Report

**DOI:** 10.3390/jcm12144569

**Published:** 2023-07-09

**Authors:** Monika Šiaudinytė, Karolina Vankevičienė, Rasa Povilaitienė, Gintautas Domža, Virginija Paliulytė, Diana Ramašauskaitė

**Affiliations:** 1 Faculty of Medicine, Vilnius University, LT-03101 Vilnius, Lithuania; 2 Vilnius University Hospital Santaros Klinikos, LT-08661 Vilnius, Lithuania; rasa.povilaitiene@santa.lt; 3 Faculty of Medicine, Institute of Clinical Medicine, Clinic of Obstetrics and Gynaecology, Vilnius University, LT-03101 Vilnius, Lithuania; gintautas.domza@santa.lt (G.D.); virginija.paliulyte@santa.lt (V.P.); diana.ramasauskaite@santa.lt (D.R.)

**Keywords:** acute abdomen in pregnant population, bowel gangrene, Meckel’s diverticulum, preterm birth

## Abstract

Acute abdomen during pregnancy is rare. Despite advances in diagnostic imaging, preoperative diagnosis in the pregnant population due to anatomical and physiological changes can pose difficulties. Diagnosis and surgery delays increase the risk of adverse outcomes for both maternal and fetal health. In symptomatic cases, explorative surgery might be essential for correct diagnosis and patient treatment. Here, we present Meckel’s diverticulum as an unusual cause of small bowel obstruction complicated with gangrene in a 34-week pregnant patient. The diagnosis was only apparent during explorative surgical laparotomy.

## 1. Introduction

Acute abdomen during pregnancy is rare and diagnosed in approximately 1 in 500 pregnant women [1]. Reasons causing acute abdomen can be both pregnancy-associated and pregnancy-unrelated. One of the most common pregnancy-unrelated acute abdomen reasons is acute appendicitis, followed by cholecystitis, intestinal obstruction, and pancreatitis [2]. Complications associated with Meckel’s diverticulum, though extremely rare, are one of the reasons causing acute abdomen during pregnancy [3]. Currently, from 1990 to 2021, 27 cases of Meckel’s diverticulum in pregnancy were published [4].

Acute abdomen diagnosis in the pregnant population can pose diagnostic challenges due to changes in anatomy and physiology during pregnancy, such as nausea, vomiting, physiological anemia, and leukocytosis [5]. However, it is important not to delay the examination and treatment of an acute abdomen during pregnancy because it increases adverse maternal and fetal outcomes [6].

While asymptomatic Meckel’s diverticulum may be left untreated, all uncertain cases or inconclusive radiological assessments should prompt surgical exploration and treatment [5]. An interdisciplinary team in managing these situations is crucial.

In this case report, we present Meckel’s diverticulum as a cause of small bowel obstruction and gangrene in the third trimester of pregnancy. The case was managed by performing laparotomy, which included necrotic bowel resection, an end-to-end ileoanastomosis, and an emergency Cesarean section at 34 weeks of gestation.

## 2. Case Report

A 36-year-old primigravida woman who was 34 weeks pregnant was admitted to the Emergency Department due to severe (10 points according to the VAS scale, even after prescribing opioids) abdominal pain and vomiting. The abdomen was tight on palpation during the general and special examination, painful throughout, and without signs of peritonitis. One of the symptoms the patient had was trapped gas and no defecation for 24 h.

The cause of the pain was unclear. The vital signs were without any concern—the pulse was 80 beats per minute, the blood pressure was 112/76 mmHg, and no fever was detected. Complete blood count and comprehensive metabolic panel laboratory tests showed no anemia or significant changes in inflammatory and biochemical parameters. The c-reactive protein was only 0.87 mg/L (0–5), and the lactate was 2.1 mmol/L (0.5–2.2). The only symptom that has led to suspicion of ileus was trapped gas and no defecation for 24 h. The gravid uterus made it difficult to perform a detailed abdominal ultrasound. An interdisciplinary team decided to do an abdominal and pelvic CT scan in order to clarify the possible etiology of acute abdominal pain. In this case, the CT scan was the first-choice diagnostic tool due to the ability to perform it urgently and obtain the results as quickly as possible. The clinical condition and pain level of the pregnant patient were evaluated as critical, so the diagnosis needed to be made immediately. The multidisciplinary team had to make a difficult decision in this particular situation whether to perform a CT scan immediately or wait for the MRI scan, but the choice was made according to the deteriorating condition of the patient.

**Figure 1:**
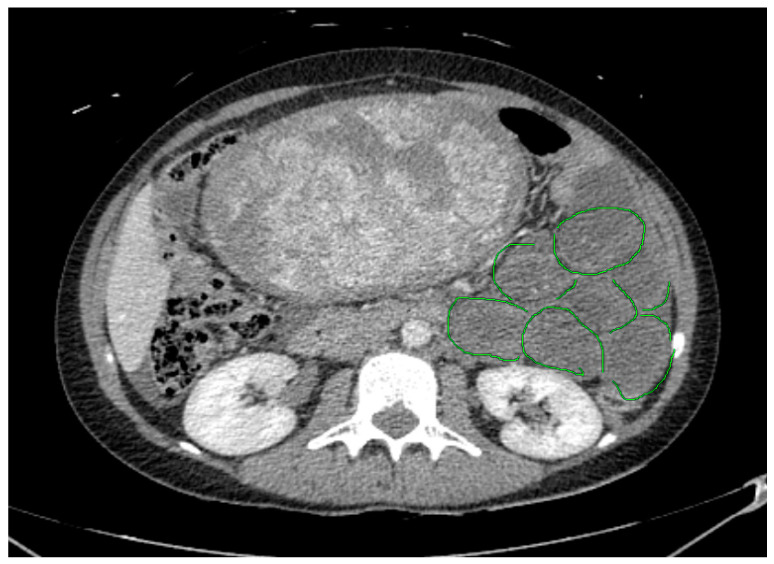
Abdomen CT findings. Dilated diameters of the small intestine locally on the patient’s left (green circles).

**Figure 2:**
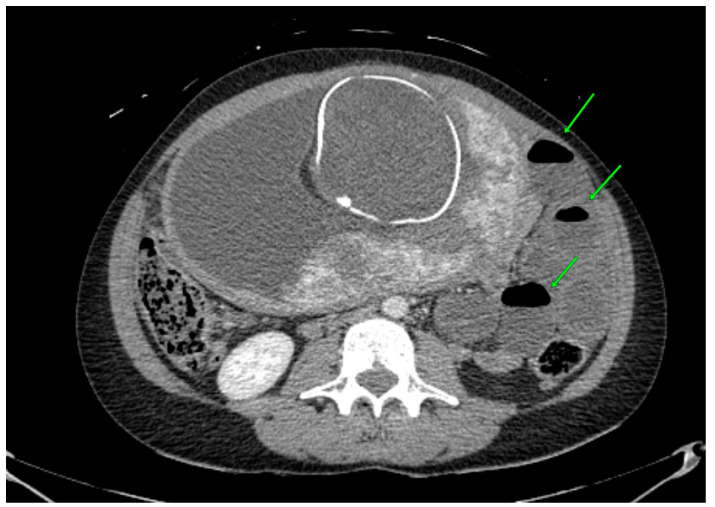
Abdomen CT findings. Air–fluid surfaces in the small intestine locally on the patient’s left—a symptom of bowel obstruction (green arrows).

The abdominal CT revealed an obstruction in the small intestine due to a probable small bowel (ileo-pelvic) reversal. Two main symptoms, a dilated diameter of the small intestine (Figure 1) and air–fluid surfaces in the intestine, were found (Figure 2).

Based on the patient’s complaints, clinical features, diagnostic tests, and preliminary diagnosis, the interdisciplinary team decided that urgent surgery for the patient’s treatment was necessary. It was decided to perform a laparotomy first to treat the ileus. In case of intestinal necrosis, a treatment plan was put in place to resect the necrotic bowels, form an end-to-end ileoanastomosis and, only if necessary, perform an emergency Cesarean section. The patient consented to the intended treatment plan.

**Figure 3:**
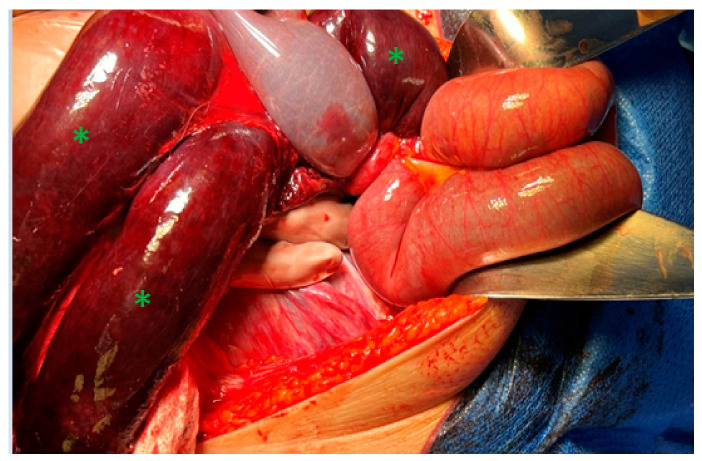
Operative findings during laparotomy. A segment of the stuck and twisted necrotic ileum was observed (green asterisks).

The surgery was performed by an experienced surgeon team—an abdominal surgeon and obstetrician-gynecologist. The surgery was started by the professor of abdominal surgery. A midline laparotomy incision under general anesthesia was performed. The laparotomy incision revealed 500 mL of hemorrhagic fluid and Meckel’s diverticulum on the left side of the abdomen, which was inflamed and “adherent” to the gravid 34-week-sized uterus. A 50 cm necrotic ileum was trapped in the “window”, which was determined by adhesions between the uterus fundus and bowels, especially Meckel’s diverticulum. Therefore, the main reason for the dynamic ileus was thought to be the adherent Meckel’s diverticulum to the uterus, which created a “window” for the obstruction of the ileum (Figure 3).

During surgery, the diverticulum was transected, and the bowel was released. The necrotic bowel was resected. As the blood supply to the distal ileum had not fully recovered and the gravid uterus made it technically impossible to inspect the remaining ileum and cecum, the decision to perform an emergency Cesarean section was made.

After an emergency Cesarean section, which was performed by an experienced senior obstetrician-gynecologist, the size of the uterus decreased, and the remaining small bowel was inspected. Its blood flow was restored, and an end-to-end ileoanastomosis was performed by the abdominal surgeon.

The newborn was delivered 51 min after the beginning of the surgery, and the Apgar scores were 3, 6, and 7 after 1, 5, and 10 min, respectively. The pH value of the umbilical artery was 7.22. During the neonatal examination hypotonus, areflexion and apnea were found. Therefore, the initial steps of newborn resuscitation were performed, and mechanical lung ventilation was started. However, it was not effective; therefore, the newborn was intubated. The newborn was immediately transported to the Neonatal Intensive Care Unit (NICU), where continuous positive airway pressure therapy (CPAP) was continued for 4 days.

The mother was treated and monitored in the Intensive Care Unit (ICU) for 2 days. Two days after the surgery, the patient had an episode of dynamic ileus, which occurred as vomiting and gastrostasis. The nasogastric probe was inserted, and 150 mL of greenish fluid was evacuated. The intravenous crystalloid infusions and parenteral nutrition were applied for four days after the surgery until the bowel function was restored and the patient started defecating. No hematochezia was noticed before the surgery or after. The mother and the newborn were discharged from the hospital after 9 days.

Afterward, histological examination revealed necrotic Meckel’s diverticulum of the small (ileum) bowel, as well as local peritonitis and segmental hemorrhagic infarction.

## 3. Discussion

Meckel’s diverticulum is present in 2% to 4% of the population, accounting for one of the most commonly diagnosed congenital malformations of the gastrointestinal tract. Its frequency is similar in both sexes, but the incidence of complications is more significant in males. Meckel’s diverticulum forms when the obliteration of the vitelline duct is incomplete. This leads to the formation of a true diverticulum of the small bowel [3]. Meckel’s diverticulum is usually composed of the typical ileal mucosa, and up to 50% of the cases also contain ectopic gastric-duodenal, pancreatic, colonic, and hepatobiliary tissue [7].

Most patients with Meckel’s diverticulum remain asymptomatic, particularly adults [8]. In the adult population, associated complications include intestinal obstruction, bowel inflammation, bleeding, neoplasm, and fistula [9,10]. Symptomatic Meckel’s diverticulum usually mimics the signs and symptoms of acute appendicitis or inflammatory bowel disease [8]. According to the literature, the most common complaints in the pregnant population with symptomatic Meckel’s diverticulum and its associated complications include abdominal pain, nausea, and vomiting. The less common complaints include abdominal distension, hematochezia, constipation, hematemesis, and diarrhea [4]. In our case, the patient’s complaints were the most common ones as described in the literature, including abdominal pain, nausea, and vomiting. However, none of them are specific and pose difficulties in differentiating the etiology of acute abdomen during pregnancy.

During pregnancy, physiological leukocytosis can mimic an acute intra-abdominal inflammatory process. According to the literature, the white blood cell counts usually revert to the pre-pregnancy levels by the sixth postpartum day. The physiological anemia during pregnancy is also observed due to increased plasma volume in proportion to the red cell volume. Physiological anemia, together with a physiological increase in heart rate, can pose difficulties in case of hemorrhage [11,12]. In this case, laboratory tests were uninformative and showed no significant changes in the complete blood panel and inflammatory parameters, notwithstanding 50 cm of intestinal necrosis and almost 500 mL of hemorrhagic fluid in the abdominal cavity.

Diagnostic imaging tools for Meckel’s diverticulum and its associated complications include abdominal ultrasound, magnetic resonance imaging (MRI), computed tomography (CT), abdominal X-ray, and endoscopy [4]. In our case, the exact cause of the acute abdominal pain was unclear. Because of the difficulties visualizing the abdominal organs using ultrasound due to a large gravid uterus, it was decided to perform a CT scan. The CT scan diagnosed local small intestine obstruction due to a probable small bowel reversal. Meckel’s diverticulum as a cause was not suspected; however, obstruction diagnosis led to a decision to perform emergency surgery and treat the patient according to the operative findings. In the systematic review, intestinal obstruction was also the most common CT finding [4].

Regarding the imaging diagnostic tools, the correct diagnosis of Meckel’s diverticulum is made during laparoscopy or laparotomy [13]. In our clinical case, neither laboratory tests nor imaging diagnostic tools have led to an accurate diagnosis. The correct diagnosis was only made on surgical exploration during laparotomy. However, the findings using diagnostic imaging tools helped to make the decision for urgent laparotomy, which was necessary for the patient’s treatment even without an accurate diagnosis.

Symptomatic Meckel’s diverticulum treatment is always a surgical resection. A pregnant patient should be managed the same as the general population. However, attention must also be paid to the well-being of the fetus. Laparoscopic treatment is considered the first choice of treatment because of its safety and effectiveness. The advantages of minimally invasive surgery include better intraoperative visualization, less pain after surgery, faster recovery, and shorter duration of hospitalization [14]. However, it can be complicated at a later gestational age due to the size of the gravid uterus. During 12 weeks of gestation, the gravid uterus enlarges to become an intra-abdominal organ. By the 20th week, the uterus reaches the umbilicus and at 36 weeks, the uterus reaches the costal margin. In order to accommodate the enlarging uterus, other intra-abdominal organs get displaced from their normal position [11,12]. Advancing pregnancy had been considered a contraindication to a laparoscopic approach, but there is growing evidence of its safety profile in all trimesters. Yet, operative laparoscopy after 20 weeks of gestation remains uncommon. Complications such as inadvertent uterine perforation during Veress needle insertion and injury during trocar placement are possible due to increased uterus size. Furthermore, operative field visualization is impaired. The potential effect of insufflation pressure on uteroplacental circulation is also considered [15].

In our case, the laparotomy approach was chosen due to the large size of the uterus in the late third trimester, almost reaching the costal margin. Despite the considerable gestational age, a Cesarean section was delayed and performed only after it was clear that the gravid uterus interfered with the removal of the twisted and gangrenous part of the intestine. According to the literature, during emergency situations, fetus delivery decisions and modes should be decided based on obstetric indications. However, delivery is indicated in every case if the continuation of the pregnancy is expected to lead to maternal morbidity or mortality. The patient should be treated with the fetus in utero if improvement of the maternal condition cannot be expected with delivery [16]. In a prospective cohort study in Italy, laparotomy performed during pregnancy was associated with Cesarean birth in more than 90% of cases [17]. In this case, it was impossible to complete the surgery and improve the maternal condition without delivering the fetus, as it was the only possible way to treat the patient.

In order to compare our case with others, we performed a selection of clinical cases in Pubmed advanced search using keywords “Meckel” and “pregnant” or “pregnancy”. A total of 10 clinical cases were included for comparison. The main characteristics of the case descriptions are presented in Table 1.

All cases were diagnosed in the second or third trimester of pregnancy; there were none in the first. The most frequently used instrumental diagnostic tool was ultrasound. MRI was chosen as a primary diagnostic tool in only one case. A CT scan as a primary diagnostic tool was chosen in one case and two cases to clarify the diagnosis after a non-informative ultrasound examination. In our case, a CT scan was also chosen after an ultrasound examination, which was not informative enough. However, we can see a trend toward a CT scan being performed less frequently due to potential radiation-related harm to the fetus. Of all these cases, Meckel’s diverticulum diagnosis was suspected preoperatively only in one case. In our case, the diagnosis was also established only during surgery. Preoperative diagnoses included a variety of reasons causing acute abdomen, including gastroenteritis, appendicitis, intestinal obstruction, intussusception, perforated ulcer, inflammatory bowel disease, and diverticulitis. In our clinical case, bowel obstruction was suspected first. In most cases, including ours, the chosen management was exploratory surgery—laparoscopy or laparotomy. Conservative treatment tactics were chosen in only three cases. One case had diagnosed Meckel’s diverticulitis; in two cases, the final diagnosis was a perforated Meckel’s diverticulum. In the presence of symptomatic Meckel’s diverticulum, even in the pregnant population, the preferred case management is surgical treatment [4].

During pregnancy, the best possible outcome must be sought not only for the mother, but also for the fetus, and every intervention must be considered and performed only when necessary and as safely as possible. Non-obstetric surgery is associated with adverse fetal outcomes such as a higher risk of prematurity, low birth weight, low Apgar scores (<7), neonatal and infant death, longer admission in the hospital, and higher medical expenses. Surgical treatment is associated with a significantly higher rate of prematurity but lower stillbirth rates in the third trimester of pregnancy than in the first one [27]. In this case, most of the reported complications related to non-obstetric surgery included low Apgar scores, prematurity, treatment in the NICU, higher medical expenses, and longer hospitalization duration. Due to the laparotomy type of surgery, it was impossible to monitor the condition of the fetus during the surgery. The main reasons for the severe condition of the newborn after birth are thought to be due to prematurity and anesthesia-induced newborn sedation. After birth, the newborn was resuscitated and treated in the NICU. Notwithstanding prematurity and resuscitation, the outcome of the newborn was good. The newborn was transferred to the ward with the mother after the condition of both the mother and the newborn were stabilized. On the discharge day, the neonatal neurological condition was consistent with gestational age.

## 4. Conclusions

Meckel’s diverticulum and its associated complications are rare causes of acute abdomen during pregnancy. In order to achieve the best outcome for both the mother and the fetus, it is crucial to manage acute abdomen cases during pregnancy with an interdisciplinary team. Despite the pregnancy, faster imaging methods and larger-scale surgery can be chosen for diagnosis and treatment. In this case, CT and laparotomy were chosen despite the higher risks for the fetus. Timely and correctly chosen diagnostic and treatment tactics led to a successful outcome for both the mother and the fetus, regardless of prematurity.

## Figures and Tables

**Table 1:** The main characteristics of case reports include Meckel’s diverticulum and the pregnant population.

Author	Year	Patient’s Age	Gestational Age during Diagnosis	Imaging	Management	Preoperative Diagnosis	Postoperative Diagnosis
Ahmed et al. [18]	2016	24	24	US ^1^ (free fluid in the abdomen with multiple septa)	Exploratory laparotomy	Appendicular perforation peritonitis	Perforated Meckel’s diverticulum
Orlandini et al. [19]	2016	24	30	US (monolateral hydronephrosis)	Exploratory laparotomy and Cesarean section	Not reported	Perforated Meckel’s diverticulum
Li et al. [20]	2017	28	30	US (signs of general small-bowel expansion and ascites). MRI scan (intraperitoneal bowel expansion with effusion)	Exploratory laparoscopy	Gastroenteritis	Small bowel obstruction
Botezatu et al. [8]	2018	30	34	US (no findings)	Clinical observation and symptomatic medication	Bowel obstruction and/or perforation	Perforated Meckel’s diverticulum
Eisdorfer et al. [21]	2018	30	22	MRI ^2^ scan	Conservative treatment	Meckel’s diverticulitis	Not reported
Hu et al. [22]	2018	28	28	US	Conservative treatment	Appendicitis	Perforated Meckel’s diverticulum
Nagata et al. [23]	2019	31	15	CT ^3^ scan (dilated small bowel loops with multiple air–fluid levels)	Emergency laparotomy	Gastroenteritis	Gangrenous Meckel’s diverticulum
Combes et al. [24]	2020	27	21	US (target sign consistent with intussusception)	Emergency laparoscopy	Intussusception	Intussusception
Beatriz Féria et al. [25]	2022	40	33	US (not informative), CT scan (showed densification of pericolic fat tissue)	Urgent Cesarean section followed by a laparotomy	Appendicitis, bowel obstruction, perforated ulcer, inflammatory bowel disease, and diverticulitis	Perforated necrotic Meckel’s diverticulum
Yantao He et al. [26]	2023	23	22	US (not informative), CT scan (left lower abdominal part of the small intestine in a vortex shape, local intestinal lumen dilated)	Exploratory laparotomy and small-bowel resection	Acute gastroenteritis and preterm labor	Meckel’s diverticulum, with a 360° twist and local necrosis without perforation

^1^ US—ultrasound; ^2^ MRI—magnetic resonance imaging; ^3^ CT—computed tomography.

## Data Availability

Data sharing is not applicable to this article.
